# Towards an Understanding of Enhanced Biomass Digestibility by *In Planta* Expression of a Family 5 Glycoside Hydrolase

**DOI:** 10.1038/s41598-017-04502-1

**Published:** 2017-06-29

**Authors:** Bryon S. Donohoe, Hui Wei, Ashutosh Mittal, Todd Shollenberger, Vladimir V. Lunin, Michael E. Himmel, Roman Brunecky

**Affiliations:** Biosciences Center, National Renewable Energy Laboratory, 15013 Denver West Parkway, Golden, Colorado, 80401 United States

## Abstract

*In planta* expression of a thermophilic endoglucanase (AcCel5A) reduces recalcitrance by creating voids and other irregularities in cell walls of *Arabidopsis thaliana* that increase enzyme accessibility without negative impacts on plant growth or cell wall composition. Our results suggest that cellulose β-1–4 linkages can be cut sparingly in the assembling wall and that these minimal changes, made at the proper time, have an impact on plant cell wall recalcitrance without negative effects on overall plant development.

## Introduction

The collective, multi-scale, chemical and physical barriers that plants have evolved to defend against microbial cellulases, often summarized in the term recalcitrance, continues to be a technological barrier to the efficient utilization of lignocellulosic biomass as a feedstock in the emerging bioeconomy^1, 2^. In the past, the idea of expressing glycoside hydrolases required for subsequent biomass hydrolysis *in planta* was proposed as part of a bio factory model of *in planta* GH expression^3–5^. There are multiple challenges that still remain with this approach including inactivation of enzymes while the plant is developing as well as cost effective extraction of the enzymes after plant senescence^5, 6^.

**Figure 1:**
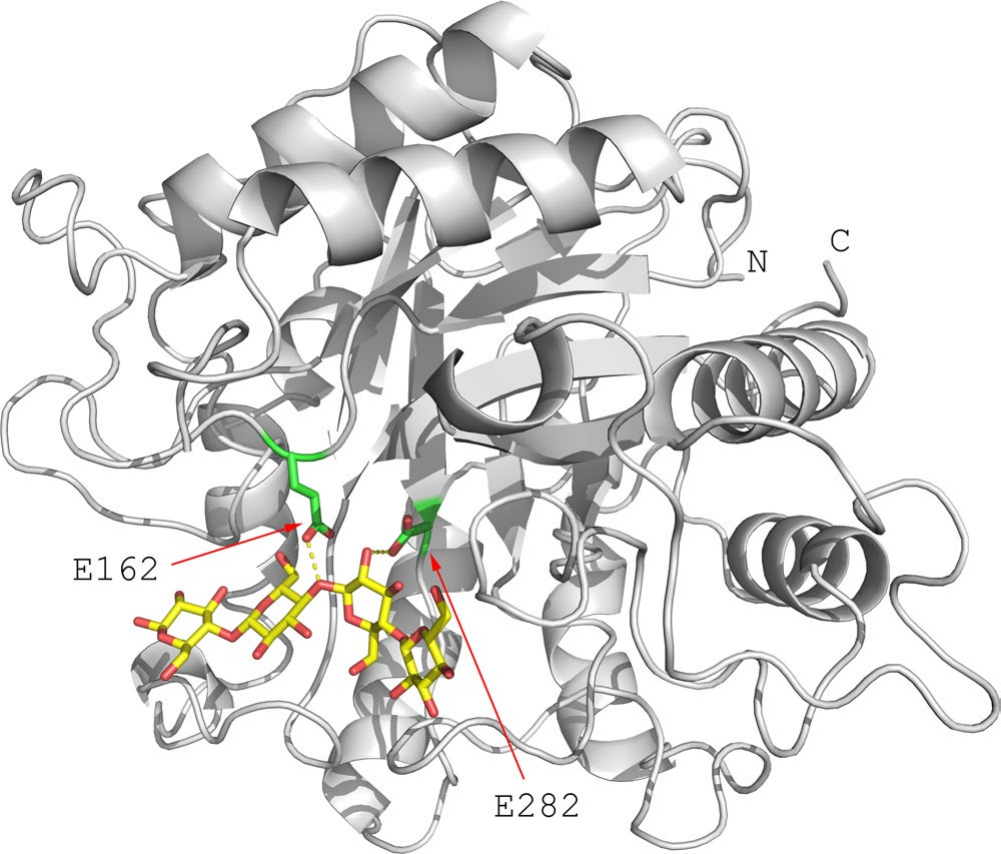
Representation of the AcCel5A (PDB ID 1ECE). A bound tetrasachharide is shown in a sticks representation with yellow carbon atoms. Two catalytic residues mutated to inactive the protein are Glu162 and Glu282 that were identified as catalytic base/acid in the original study^34^ are shown as sticks with green carbon atoms.

Several ongoing efforts seek to reduce biomass recalcitrance through the direct modification of plant cell wall biosynthetic pathways, often with unintended and negative consequences for plant growth and viability^7, 8^. Another approach, and the one explored in this work, is to introduce exogenous proteins that are capable of modifying cell walls^9, 10^. This basic approach has been applied to reducing xylan acetylation^11, 12^, modifying ferulic acid linkages^13, 14^, and pectins^15^. In this work, we utilize the hyper-thermophilic (T_opt_ 81^o^C) glycoside hydrolase family 5 endocellulase, AcCel5A (formerly E1), from *Acidothermus cellulolyticus*. AcCel5A is a well-studied and characterized enzyme, a hyperthermophilic endoglucanase that creates nicks in cellulose, and as part of biomass degrading enzyme cocktails synergizes with exo-glucanases^16, 17^. AcCel5A has been expressed in a variety of plants including maize, tobacco, potato, rice and duckweed, sometimes with deleterious effects^18–21^. Our AcCel5A construct lacks the CBM found in the native enzyme which may impact association with cellulose however, the catalytic domain is still active^16, 22, 23^. We have shown previously that *in planta* expression of low levels of AcCel5A reduces the recalcitrance of plant cell walls, making them more amenable to conversion; however, the mechanism of this recalcitrance reducing plant biomass modification remains unknown^24^. It was also shown that the reduction in recalcitrance could not be recapitulated by simply adding AcCel5A enzymes exogenously to plant tissue^24^. We hypothesize that the observed digestibility enhancement must rely on a complex interaction between the endoglucanase and the developing cell wall, for example improving accessibility to the microfibrils by creating nicks and voids in the plant cell wall that an exogenously added enzyme cocktail with restricted access cannot replicate. To elucidate this mechanism of recalcitrance reduction, we have generated two AcCel5A expressing Arabidopsis lines, the first with a catalytically active AcCel5A and the second with a catalytically inactive AcCel5A (AcCel5A-D). The AcCel5A-D line was designed to help us address whether or not AcCel5A catalytic activity is required or if simply the physical presence of AcCel5A interacting with the developing plant cell wall is sufficient to alter walls enough to reduce recalcitrance (Fig. 1). Here, we show that *in planta* expression of AcCel5A under a constitutive promoter reduces recalcitrance by creating dispersed voids and delamination within cell walls; as well as general surface irregularities that increase available surface area and subsequently, hydrolytic enzyme accessibility. The negative impacts on plant growth, cell wall carbohydrate composition, and cellulose structure often encountered with *in planta* expression of enzymes were not observed.

## Results

### AcCel5A expression in Arabidopsis reduces cell wall recalcitrance

**Figure 2:**
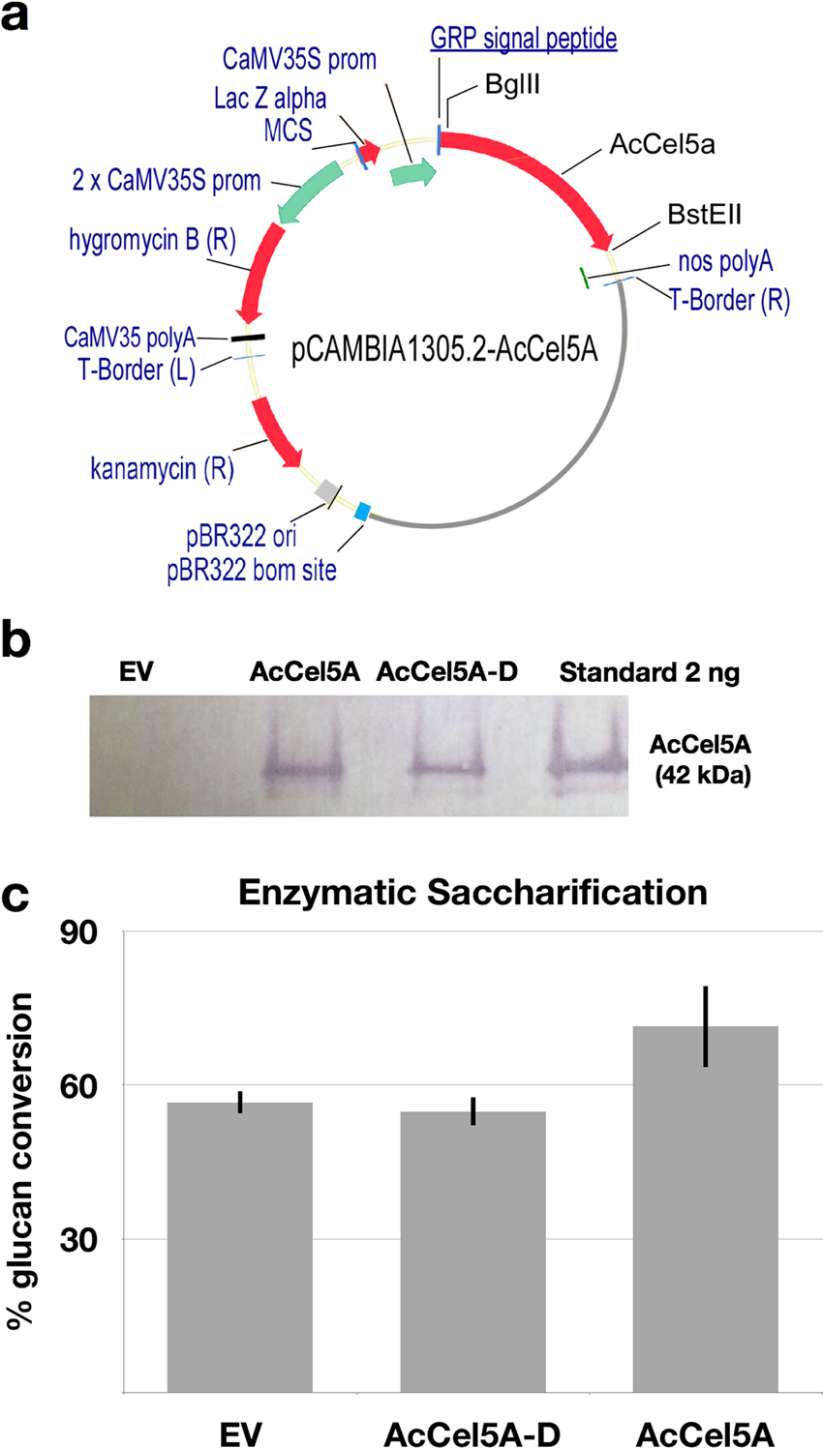
*Acidothermus cellulolyticus* endocellulase AcCel5A expression construct, AcCel5A Western blot, and enzymatic saccharification. (**a**) The backbone vector pCAMBIA1305.2 (www.cambia.org) has a glycine-rich protein (GRP) signal peptide for extracellular targeting. The catalytically active AcCel5A gene (see supplemental material 1) or AcCel5A catalytic dead gene (AcCel5A-D) replaced the catalase intron-GusPlus gene cassette and the terminator was nopaline synthase (nos) polyA. See Materials and Methods for details. (**b**) Western blot of wild-type empty vector control (EV), AcCel5A and AcCel5A catalytically inactive (AcCel5A-D) transgenic plant lines showing the presence or absence of the AcCel5A protein in plant stem tissue, and a standard of purified AcCel5A. (**c**) Enzymatic saccharification. AcCel5A expression in plant cell walls increases the % glucan conversion by approximately 25% over control levels. The AcCel5A-D data is from line 37-4-a and the AcCel5A data is from line 21-2-b.

Expressing AcCel5A within the developing plant cell wall partially circumvents the natural barriers that plants have evolved to counteract microbial cellulases. The AcCel5A (n = 5) expressing plants have a 25% increase in glucan conversion compared to the empty vector (EV) control (n = 6) or the AcCel5A-D (n = 8) plants (Fig. 2c). The observation that the AcCel5A-D mutant has no effect on reducing recalcitrance is not surprising, but it does answer the question of whether or not the mere presence and binding of AcCel5A to the cell wall without catalytic activity is sufficient to disrupt cell wall architecture and improve enzymatic saccharification. In essence, an active AcCel5A is required to achieve this effect.

### AcCel5A expression in Arabidopsis does not impact growth, cell wall composition, or cellulose crystallinity

**Figure 3:**
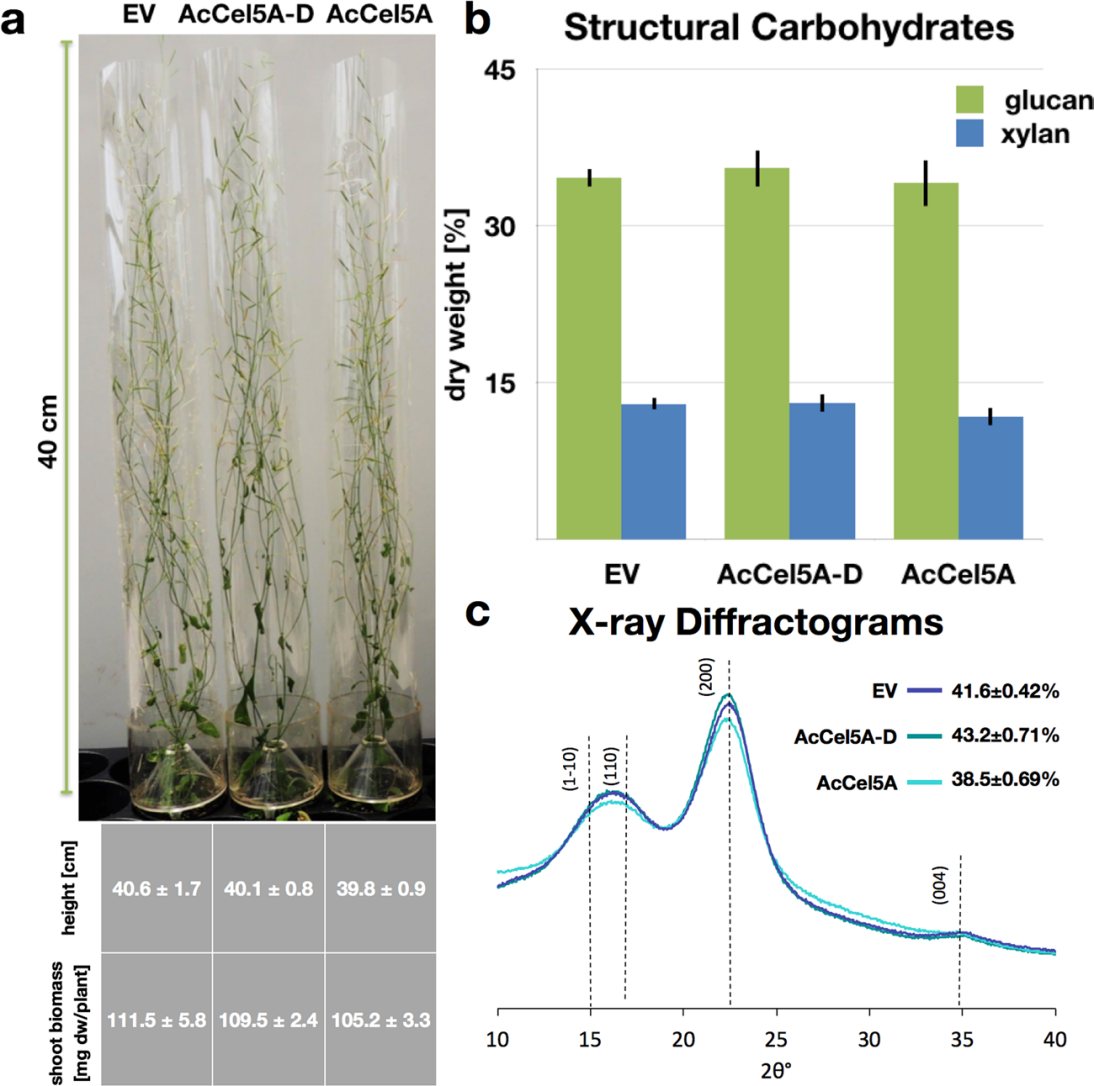
Plant growth, biomass accumulation, structural carbohydrate composition, and cellulose crystallinity. (**a**) Plant height and shoot biomass dry weight (dw) of wild-type empty vector control (EV), AcCel5A catalytically inactive (AcCel5A-D), and transgenic AcCel5A lines at a mid-senescence growth stage, ten weeks after germination. Values are presented as the mean (±SEM) of six measured plants. There are no significant differences in plant heights or masses among the plant lines. (**b**) Structural carbohydrate chemical composition shows no significant differences in major carbohydrate cell wall polymers among the three plant lines. (**c**) X-ray diffractograms from wild-type empty vector control (EV), AcCel5A catalytically inactive (AcCel5A-D), and transgenic AcCel5A stem samples. The crystallinity index values (CI) derived from the spectra are indicated. The AcCel5A-D data is from line 37-4-a and the AcCel5A data is from line 21-2-b.

Both the active AcCel5A and catalytically inactive AcCel5A-D are expressed *in planta* at low levels. Approximately 0.3 ng AcCel5A/mg dry biomass in the AcCel5A plants, 0.24 ng AcCel5A-D/ mg dry biomass in the AcCel5A-D plants, and no detectable AcCel5A protein in the EV plants as shown in the Western blot (Fig. 2b). All three lines, the transformed wild-type empty vector control (EV), AcCel5A catalytically inactive (AcCel5A-D), and transgenic AcCel5A lines of *A. thaliana* grew with a normal overall plant phenotype (Fig. 3a). At the time of harvest, the senesced plant lines had very similar shoot height; as well as shoot mass per plant (Fig. 3a). The bulk cell wall glucan and xylan composition of AcCel5A (n = 5) and AcCel5A-D (n = 8) transformed plants were analyzed and are approximately 34% glucan and 12% xylan, which is nearly identical to the EV composition (n = 6), showing that there is no change in glucan or xylan content in cell walls of the transformed plants (Fig. 3b). These are important findings for bioconversion research because losses in plant biomass, or losses of fermentable carbohydrates, are obviously undesirable traits for future bioenergy crops. We have also examined cellulose crystallinity, and like the other phenotypic characterizations, again see no significant differences in crystallinity among the plants tested. In Fig. 3c, the X-ray diffraction (XRD) diffractograms of the samples are similar, indicating that cellulose crystalline content remained nearly unaltered. This phenotypic characterization suggests that the reduction in recalcitrance accompanying integration of active AcCel5A is not caused by a change in bulk cell wall chemical composition or a change in cellulose crystallinity, and yet has no deleterious effect on plant growth or biomass accumulation.

### *In-planta* expression of AcCel5A reduces recalcitrance by creating dispersed voids in cell walls

**Figure 4:**
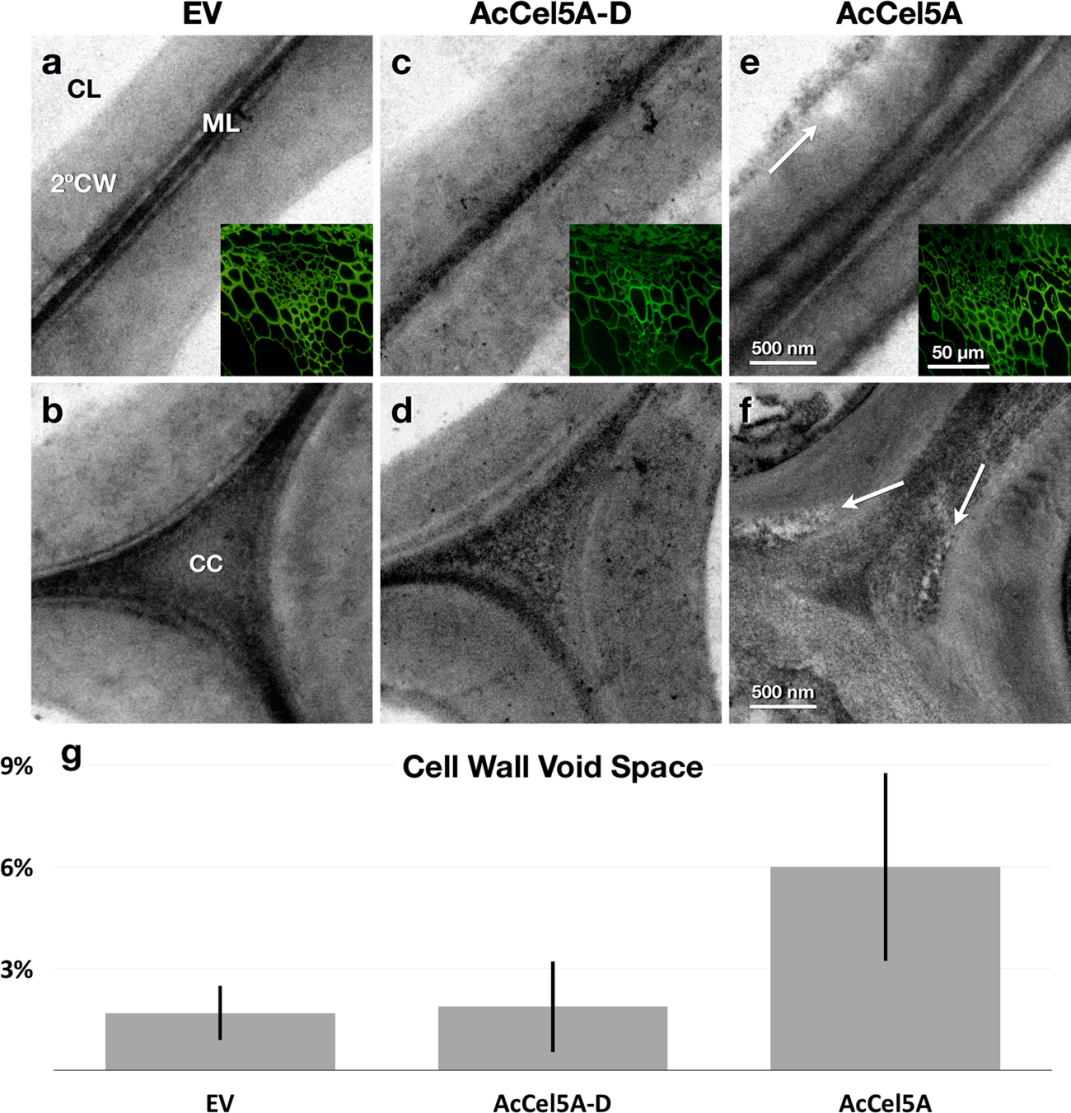
Microscopic analysis of cell walls. Transmission electron micrographs (TEM) of interfascicular fiber cell walls and confocal scanning laser micrographs (CSLM) of vascular bundles sectioned and images in transverse section. The confocal scanning laser micrographs CSLM images (insets) of the empty vector control (**a**), AcCel5A catalytically inactive (**c**), and AcCel5A expressing (**f**) plants display largely the same cell and tissue morphology around the vascular bundles with no obvious evidence for xylem collapse or occlusion, or other signs of cell wall weakening. The TEM micrographs; however, reveal differences among the samples. The AcCel5A expressing cell walls displayed more irregular surfaces and regions of clearing or low density (**e**,**f** arrows). The % void space within the cell walls was measured by image analysis of the TEM micrographs are presented (**g**). Error bars show standard deviation. CL-cell lumen, 2°CW-secondary cell wall, ML-middle lamella, CC-cell corner. The AcCel5A-D data is from line 37-4-a and the AcCel5A data is from line 21-2-b.

**Figure 5:**
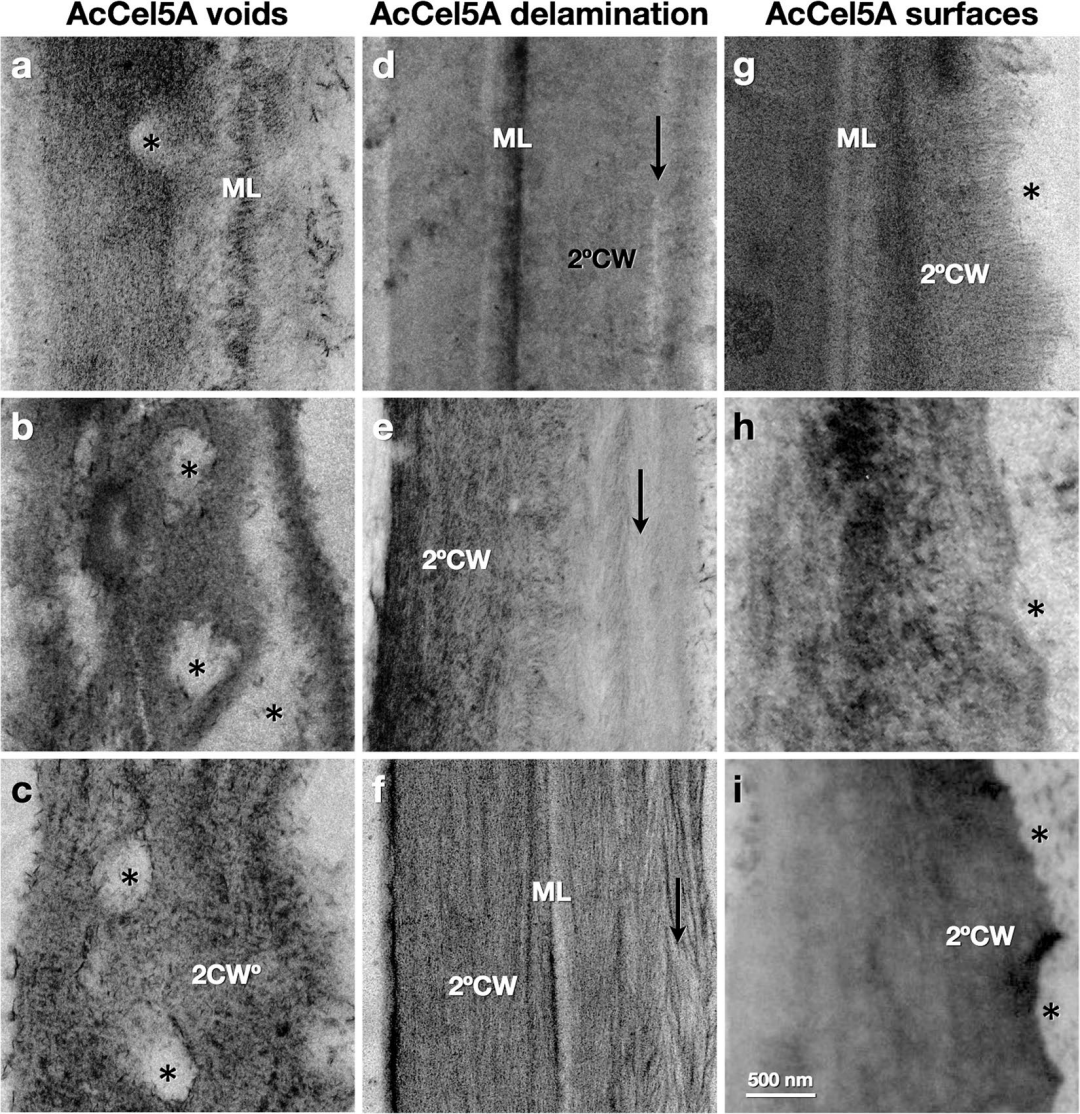
Gallery of TEM micrographs of the AcCel5A expressing plant cell walls of interfascicular fiber cells display examples of cell wall disruption morphologies. The tissue and cell orientation is the same as in Fig. 4 with sections transverse to the growth axis of the plant. These micrographs reveal some of the more extreme examples of voids or clearing zones (*, **a–c**), wall delamination (arrows, **d–f**), and irregular, scalloped wall surfaces (*, **g–i**). ML-middle lamella, 2°CW-secondary cell wall. The e AcCel5A data is from line 21-2-b.

To further investigate possible mechanisms underlying the increased glucan conversion observed, we performed microscopic analysis of these cell walls. Plants from two lines of each of the three samples (EV, AcCel5A-D, and AcCel5A), and multiple stem sections from each plant were processed. 4–12 confocal scanning laser micrographs confocal scanning laser microscopy (CSLM) and 40–45 transmission electron micrographs (TEM) were captured and visually inspected for each sample (306 micrographs in total). The CSLM images (Fig. 4 insets) of the EV, AcCel5A-D, and AcCel5A stems display largely the same cell and tissue scale morphology surrounding the vascular tissue, with no signs of xylem collapse or occlusion, or other evidence of systemic cell wall weakening. These cellular defects would have likely led to a dwarfed growth phenotype. When we subjected the plants to TEM imaging; however, we observed several morphological differences between the transgenic AcCel5A expressing and control plant cell walls. We observe that the plant cell walls in the AcCel5A transgenic plants have multiple clearing zones of various sizes dispersed along their secondary walls. In some cases, these voids have expanded to create physical ruptures of the plant cell wall (Figs 4e,f and 5a,b). These voids were not observed in the EV or AcCel5A-D plants (Fig. 4a–d). Furthermore, some walls of the AcCel5A plants also displayed a striated morphology, suggesting partial delamination (Fig. 5c,d). Another morphology observed in some AcCel5A transgenic plant cell walls was a scalloping of the lumenal cell wall surfaces, which was reminiscent of cell walls from plant biomass that had been digested by cellulase cocktails (Fig. 5e,f). The pattern of both the clearing zones and the surface erosion may be the result of the concentrated delivery of AcCel5A. Each of these cell wall morphologies resulted in an increase in the surface area accessible for subsequent enzymatic digestion. Increasing cell wall accessibility to cellulase enzymes is the critical objective of all thermochemical and mechanical pretreatment methodologies used today^7^. Indeed, some features commonly seen in pretreated plant material are strikingly similar to the AcCel5A-plant material generated here; and the cell wall void space measured in the AcCel5A cell walls is comparable to what has been measured previously in dilute acid pretreated material^7^.

## Discussion

### Modulated endoglucanase activity allows for reduction of recalcitrance while maintaining normal plant growth

Recently, expression in tobacco of a similar, but not hyper-thermophilic endoglucanase from *Trichoderma reesei*, TrCel5A, has been reported^25^. When TrCel5A is constitutively expressed, it causes defects in plant growth, including reduced stem length and an asymmetrical curly leaf phenotype. In addition, TrCel5A transformed plants had significantly reduced cellulose crystallinity and the glucan/xylan ratio was altered. We suspect that if AcCel5A was expressed at high titers and fully active during early plant development, it too would likely result in undesirable phenotypes and altered chemical and physical properties of the cell wall polymers. AcCel5A; however, is a hyperthermophilic enzyme with low activity at normal plant growth temperatures. This is a critical characteristic that likely permits its incorporation into cell walls where is reduces recalcitrance, while maintaining normal growth. Also, if this moderate endoglucanase activity, conferred by an enzyme operating at suboptimal conditions, is the key to achieving recalcitrance reduction without a reduction in biomass accumulation, it may be possible to attenuate the production or activity of other enzymes, such as TrCel5A, to achieve similar outcomes. Furthermore our AcCel5A constructs lack a carbohydrate binding module (CBM), which further reduces activity, while the TrCel5A has a CBM. Whereas excessive endoglucanase activity presumably disrupts plant cell wall architecture enough to lead to abnormal growth phenotypes, carefully modulated endoglucanase activity allows for reduction of recalcitrance, while maintaining normal growth phenotypes. We suspect that the correct level of endoglucanase activity generates a subtle, dispersed modification of the cell wall architecture over time.

## Materials and Methods

### Gene synthesis and transformation constructs

For cloning AcCel5A into the vector, pCAMBIA1305.2, the AcCel5A gene sequence coding the catalytic domain (CD) was codon-optimized for expression in Arabidopsis and then synthesized by GenScript (Piscataway, NJ), with agatctg (BglII and an additional g nucleotide to keep the codon in frame) added at the 5′ end, and taaggtgacc (three additional nucleotides and BstEII) at the 3′ end (Fig. 1). The resultant gene sequence was ligated to BglII-BstEII cut pCAMBIA1305.2 (GenBank Accession No. AF354046; with a BglII cut at vector position 82, and BstEII cut at position 2133). The pCambia1305.2-AcCel5A binary vector confers hygromycin B resistance as selection marker, with AcCel5A expression driven by the cauliflower mosaic virus 35 S promoter (CaMV35S). The construct for the catalytically inactive AcCel5A was based on pCAMBIA1305.2-AcCel5A with mutagenesis of two amino acids (E162A and E282A) (Fig. 1) in the catalytic site to abolish activity was prepared by GenScript (Piscataway, NJ) - the resulting vector was named pCambia1305.2-AcCel5A-D (Fig. 1a). The construct for the empty vector control (WT-EV) (i.e., pCAMBIA1305-Gus minus) was built by deleting the fragment of CaMV35S promoter and the GusPlus gene (position 11303–2054) from pCAMBIA1305.1 (GenBank accession no. AF354045.1).

The original AcCel5A (http://www.uniprot.org/uniprot/P54583) has its own signal peptide (position 1-41 aa), followed by the catalytic domain (position 42–400 aa) and CBM2 (position 458–562 aa). In this study, the AcCel5A’s native signal peptide is replaced with rice glycine rich protein (GRP) signal peptide encoded by the nucleotide sequence in the vector pCambia1305.2; GRP signal peptide is 27 amino acid in length (see supplemental data file, sequence 2; sequence highlighted in grey shade). AcCel5A’s original CBM2 domain was removed, with only the catalytic domain was included for expression in the expression cassette (see supplemental data file, sequence 2; sequence highlighted in yellow shade).

### Agrobacterium-mediated transformation and transgenic plant generation

The AcCel5A construct (pCAMBIA1305.2-AcCel5A), the AcCel5A-D construct (pCAMBIA1305.2-AcCel5A-D), and the empty vector (pCAMBIA1305-Gus minus) were separately introduced into *A. tumefaciens* strain C58 competent cells of using a freeze-thaw method^26^. Kanamycin (50 µg/mL) and rifampicin (10 µg/mL) were used as selection markers for positive colonies. The positive colonies were confirmed by PCR analysis for the presence of the target vectors. The floral dip transformation method^27^ was used to transform *Arabidopsis thaliana* Col-0 with *A. tumefaciens* C58 strains harboring the above target constructs. The transformed plants were grown to maturation for T1 seed collection. These seeds were germinated and used for further selection of homozygous transformants as described previously^28^.

### AcCel5A and AcCel5A-D transgenic Arabidopsis plants

In this study, we have generated two types of AcCel5A expressing Arabidopsis lines, the first with a normal, catalytically active AcCel5A, and the second with a catalytically inactive AcCel5A (referred as AcCel5A-D). Nine independent transformed T1 Arabidopsis AcCel5A and AcCel5A-D transgenic lines were generated, respectively. Total RNA was extracted from these transgenic lines and was reverse-transcribed to cDNA. The prepared cDNA and the primers (421.1 F, GCACAAAGGTATAAGGGAAATCCA; 421.1 R, CCAGCCCTTTCTGCAGCTAA; amplicon size: 125 bp) were used for the real-time RT PCR analysis, which detected the target transcripts in the nine AcCel5A and AcCel5A-D transgenic lines.

Of these nine AcCel5A transformants, two transformants showed nearly the same glucose release after enzymatic saccharification, and were about 15% higher than that of the empty vector (EV) control or the AcCel5A-D, thus were selected to further process to their T3 generation, for which their homozygosity was confirmed by segregation analysis. These two AcCel5A lines were referred as lines 21-2-b and 21-3-c respectively, and were used in the enzymatic saccharification, structural carbohydrate determination, and the imaging analyses, from which the representative data were presented.

In contrast, of the nine AcCel5A-D transformants there were no significant differences in their glucose release among these nine AcCel5A-D lines, and also no significant difference from the EV control, after enzymatic saccharification. Two AcCel5A-D transformants were selected to further process to their T3 generation to confirm their homozygosity through segregation analysis. These were referred to as lines 37-4-a and 37-8-a respectively. These two lines were used as controls in the enzymatic saccharification, structural carbohydrate determination and the imaging analyses, and the representative data were presented.

### Plant growth, harvesting and milling

*Arabidopsis thaliana, ecotype Columbia*-0 (Col-0) was used as the parent line for *Agrobacterium*-mediated transformation with the AcCel5A gene constructs. Col-0 seeds were germinated on ½ MS agar medium containing 1% sucrose. After 2-d incubation for cold stratification at 4 °C, the medium plates were kept in a growth chamber with 16 h light (140 µmol E m^−2^ s^−1^) and 8 h dark cycles at 24 °C for 5 d, then the seedlings were transferred to pots that contained Metro-Mix 360 soil (SunGro Horticulture, Canada) and kept under light shelves of ArabiSun Lighting System (Lehle seeds, Texas, USA) with 16 h light (170 µmol E m^−2^ s^−1^) and 8 h dark cycles at 24 °C. Transgenic and empty vector control seedlings were randomly placed in the greenhouse and watered with distilled H_2_O twice a week, with 16 h light (200 to 300 µmol E m^−2^ s^−1^), 8 h dark cycles at 24 °C. Plants were harvested at the senescent stage as described previously^28^. Briefly, the naturally dried shoot samples were then knife-milled and passed through a 20-mesh (1 mm) screen twice for uniformity (Wiley knife mill; Thomas-Wiley, Philadelphia).

### Western blotting

Five mg of ground senesced plant material was extracted using 70 µL of lithium dodecyl sulfate (LDS) sample buffer and run on SDS-PAGE gel and blotted with anti-AcCel5A antibody^24, 29^ to confirm the expression of the AcCel5A catalytically dead (AcCel5A-D) and AcCel5A proteins. Standards were included with 2, 5, 10, and 20 ng quantities of AcCel5A to approximate AcCel5A expression levels. The expression level of AcCel5A was ~1.5 ng and that of the AcCel5A-D was ~1.2 ng.

### Structural carbohydrate determination and cellulose crystallinity

Milled samples were wrapped in teabags, treated with alpha-amylase (Spirizyme Ultra – 0.25%) and β-D-glucosidase (Liquozyme SC DS – 1.5%) (Novozyme) in 0.1 M sodium acetate buffer (24 h, 55 °C, pH 5.0) to remove starch and cellobiose (16 mL enzyme solution per one g biomass), then extracted with 96% ethanol (20 + /− 4 h). Structural carbohydrates were determined utilizing NREL laboratory analytic procedure (LAP)^30^. Briefly, Arabidopsis samples were destarched, weighed, and then subject to a two-stage sulfuric acid hydrolysis. Glucose, xylose, galactose, arabinose and mannose were determined by HPLC analysis. The crystallinity indexes (CI) of biomass samples were measured by X-ray diffraction (XRD) using a Rigaku (Tokyo, Japan) Ultima IV diffractometer with CuKα radiation having a wavelength λ(Kα1) = 0.15406 nm generated at 40 kV and 44 mA. The diffraction intensities of dried samples placed on a quartz substrate were measured in the range of 8 to 42° 2θ using a step size of 0.02° at a rate of 2°/min. The crystallinity indexes (CrI) of the biomass samples were calculated according to the method using eq. 1 described by Segal *et al*.:

1 $$CrI=\frac{{I}_{200}-{I}_{Am}}{{I}_{200}}$$

where I_200_ and I_Am_ are the maximum and minimum intensity of diffraction at approximately 2θ = 22.4–22.5° and 2θ = 18.0–19.0°, respectively^31^.

### Enzymatic saccharification biomass recalcitrance assay

Milled Arabidopsis biomass samples were individually hand weighed to 5 mg (+/−3%) and then placed into a 96-well Hastelloy reactor plate. The wells were filled with 200 µL of deionized water, sealed with 3 M brand Teflon tape and left to incubate at 50 °C overnight for approximately 16 to 17 h to fully hydrate. The Hastelloy plates were then taken out and cooled to room temperature before being heated to 180 °C at 130 psi for 12.5 min in a 2 gallon Parr rector (model 2550). These sample plates were then rapidly cooled with water and centrifuged at 1700 rpm for 20 min. Following centrifugation, 40 µL of Novozymes Cellic CTEC2 enzyme diluted to 8.75 mg/mL was added, and the sample plates were incubated for 70 h at 50 °C. Samples were then removed, allowed to cool to RT before centrifuging again at 1700 rpm for 20 min. The samples were then prepared at a 1:100 final dilution into Megazyme Gopod (glucose oxidase/peroxidase) and XDH (xylose dehydrogenase) colorimetric assays to determine their D-glucose and by UV spectrophotometry. Calculation of glucose conversion yield was determined by taking the average mass of biomass measured in each sample well, calculating the mg glucan content by multiplication, and then taking the glucan liberated by the digestion and diving by the glucan present in the sample to yield a percent conversion.

### Sample preparation and microscopic analysis

Arabidopsis infloresence stems were hand sectioned under a dissection steremicroscpe to fit into 0.2mm deep planchettes and surrounded by 0.15 M sucrose solution as a cryoprotectant^32, 33^. The sample were then cryo-preserved by high-pressure freezing in a Leica EMPact2 (Leica, Wetzlar, Germany). Cryo-preserved samples were then placed in a cryo-tube vials and transferred into vials containing one mL aliquots 2.5% glutaraldehyde / 0.1% uranyl acetate in dry acetone and freeze-substituted with the following temperature regime: −90 °C for 72 h, ramp to −30 °C over 3 h, hold at −30 °C for 21 h, ramp to 3 °C over 3 h, hold at 3 °C for 21 h, ramp to 24 °C over 3 h, hold at 24 °C for ~1 h, rinse 3X in dry acetone at RT. Samples were removed from the planchets and infiltrated in a graded series (7.5%, 15%, 30%, 60%, 90%, 3X 100%) of LR White resin over 3 d. Samples were transferred into Easy Molds for polymerization and manually oriented to ensure that transverse sections can be easily cut. Resin was polymerized for 48 h in a vacuum oven at 60 °C.

For confocal scanning laser microscopy (CSLM), semi-thin transverse sectioned samples were positioned on glass microscope slides and stained with 0.1% acriflavine in water. Images were captured using a 60X 1.4NA Plan Apo lenses on a Nikon C1 Plus microscope (Nikon, Tokyo, Japan), equipped with the Nikon C1 confocal system using the Argon tunable laser at 488 nm, and operated via Nikon’s EZ-C1 software. For transmission electron microscopic (TEM) analysis, LR White embedded stem samples were transverse sectioned to ~60 nm with a Diatome diamond knife on a Leica EM UTC ultramicrotome (Leica, Wetzlar, Germany). Sections were collected on 0.5% (v/v) Formvar-coated slot grids (SPI Supplies, West Chester, PA). All grids were post-stained for four min with 2% (w/v) aqueous uranyl acetate and 2 min with Reynolds lead citrate. Images were taken with a four mega-pixel Gatan UltraScan 1000 camera (Gatan, Pleasanton, CA) on a FEI Tecnai G2 20 Twin 200 kV LaB6 TEM (FEI, Hilsboro, OR). To quantify the void space created within cell walls by AcCel5A, TEM images were processed to threshold intra-wall void spaces. Six ROIs from six different images taken of sections from two blocks of two plants from each of the three samples were analyzed. Thresholds were determined based on the mean and standard deviation of pixel values within the cell lumen. The threshold was then set to two standard deviations from the mean value of the cell lumen.

## Electronic supplementary material

Supplementary data
