# Supplementary material for: Towards an Understanding of Enhanced Biomass Digestibility by In Planta Expression of a Family 5 Glycoside Hydrolase
Source: Sci Rep. 2017 Jun 29;7:4389. doi: 10.1038/s41598-017-04502-1 (PMC5491509; doi:10.1038/s41598-017-04502-1)
Supplement: Supplementary file 1 — Supplementary data [file 41598_2017_4502_MOESM1_ESM.pdf]

# Towards an Understanding of Enhanced Biomass Digestibility by *In Planta* Expression of a Family 5 Glycoside Hydrolase

Bryon S. Donohoe, Hui Wei, Ashutosh Mittal, Todd S. Shollenberger, Vladimir V. Lunin
Michael E. Himmel, and Roman Brunecky*

**Supplementary Data**

**Figure S1.** Codon optimized AcCel5a gene sequence with the BglII and BstEII at the 5’ and 3’ ends respectively:

(1) 5’ end: AGATCTG; BglII recognizes the sequence A^GATC_T, then one additional nucleotide (G) added to register with the codon.

(2) 3’ end: TAAGGTGACC; two stop codes, and BstEII cut site (recognizing G^GTNAC_C).

(3) In between, it is the AcCel5a coding sequence.

*AGATCTG*GCAGGAGGTGGTTATTGGCACACAAGTGGTAGAGAGATACTCGATGCAAACAACGTTCCAG
TGAGGATAGCAGGAATCAACTGGTTCGGATTTGAAACATGTAATTACGTTGTGCATGGTTTGTGGTCAA
GAGATTACAGGAGTATGCTCGATCAAATCAAGAGTCTTGGATACAACACAATTAGATTACCTTACTCTGA
TGATATACTTAAACCTGGTACTATGCCAAACTCTATTAATTTCTACCAGATGAATCAGGATCTCCAAGGAC
TCACTAGTCTTCAAGTTATGGATAAGATAGTGGCTTATGCAGGACAGATTGGTTTGAGAATTATCCTCGA
TAGACACAGGCCTGATTGCAGTGGACAGTCTGCTCTCTGGTACACTTCTTCAGTTTCAGAAGCAACATGG
ATTAGTGATTTGCAGGCTCTCGCACAAAGGTATAAGGGAAATCCAACAGTTGTGGGTTTTGATCTTCATA
ACGAGCCTCACGATCCAGCTTGTTGGGGATGCGGAGATCCTTCTATTGATTGGAGATTAGCTGCAGAAA
GGGCTGGAAACGCAGTTCTCTCAGTGAATCCAAACCTTTTGATCTTCGTTGAGGGAGTGCAATCTTATAA
CGGAGATTCATACTGGTGGGGTGGTAACTTGCAGGGAGCTGGTCAATACCCTGTTGTGTTGAATGTTCC
AAACAGACTCGTGTATTCTGCTCATGATTACGCAACTTCAGTTTATCCTCAAACATGGTTTTCTGATCCTA
CCTTCCCAAATAACATGCCAGGAATCTGGAATAAGAACTGGGGTTATCTTTTTAATCAGAACATAGCTCC
AGTTTGGTTGGGAGAGTTCGGTACTACACTCCAGTCTACCACTGATCAAACATGGTTAAAAACCCTTGTG
CAGTACCTTAGGCCTACAGCTCAATATGGAGCAGATTCATTTCAGTGGACCTTCTGGAGTTGGAATCCAG
ATTCTGGAGATACCGGAGGTATCTTGAAGGATGATTGGCAAACTGTTGATACCGTGAAAGATGGTTATT
TGGCTCCTATTAAGAGTAGTATATTTGATCCTGTGGGTATGTGA*TAAGGTGACC*

**Figure S2.** Amino acid sequence for the expressed AcCel5a catalytic domain (highlighted yellow) that preceded the rice glycine rich protein (GRP) signal peptide (highlighted grey).

MATTKHLALAILVLLSIGMTTSARTLLDLAGGGYWHTSGR

EILDANNVPVRIAGINWFGFETCNYVVHGLWSRDYRSMLD

QIKSLGYNTIRLPYSDDILKPGTMPNSINFYQMNQDLQGL

TSLQVMDKIVAYAGQIGLRIILDRHRPDCSGQSALWYTSS

VSEATWISDLQALAQRYKGNPTVVGFDLHNEPHDPACWGC

GDPSIDWRLAAERAGNAVLSVNPNLLIFVEGVQSYNGDSY

WWGGNLQGAGQYPVVLNVPNRLVYSAHDYATSVYPQTWFS

DPTFPNNMPGIWNKNWGYLFNQNIAPVWLGEFGTTLQSTT

DQTWLKTLVQYLRPTAQYGADSFQWTFWSWNPDSGDTGGI

LKDDWQTVDTVKDGYLAPIKSSIFDPVGM

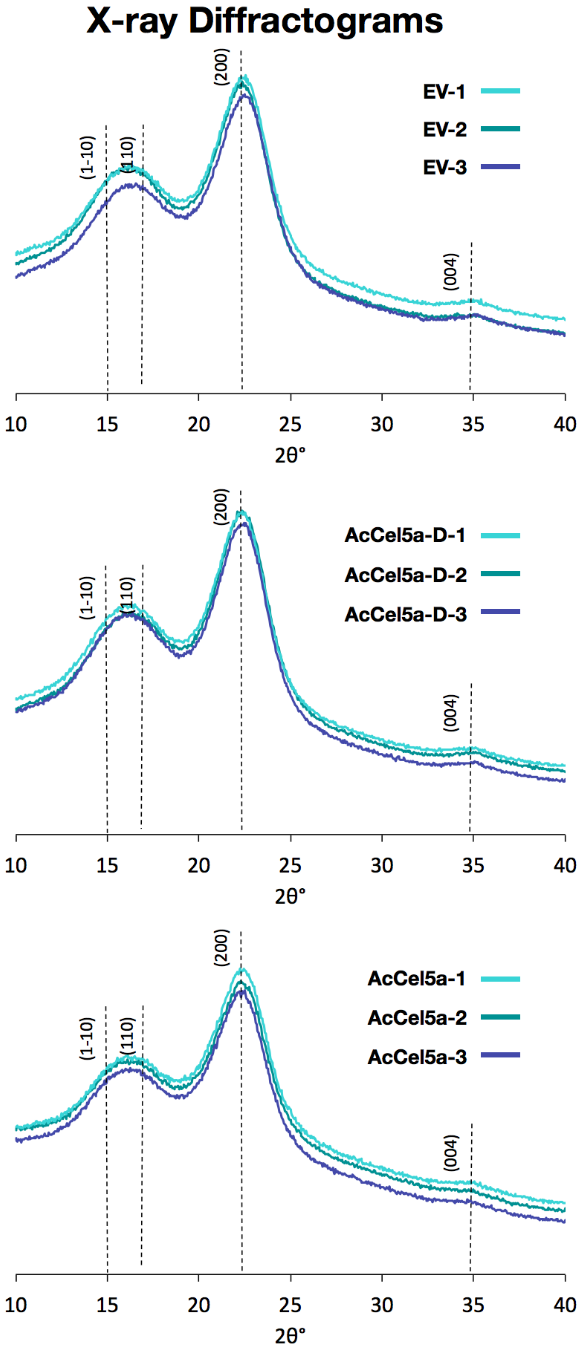


**Figure S3.** X-ray diffractograms from wild-type empty vector control (EV), AcCel5a catalytically inactive (AcCel5a-D), and transgenic AcCel5a stem samples. For each set of triplicate experiments the spectra have been artificially offset in the vertical axis for visualization. The AcCel5A-D data is from line 37-4-a and the AcCel5A data is from line 21-2-b.

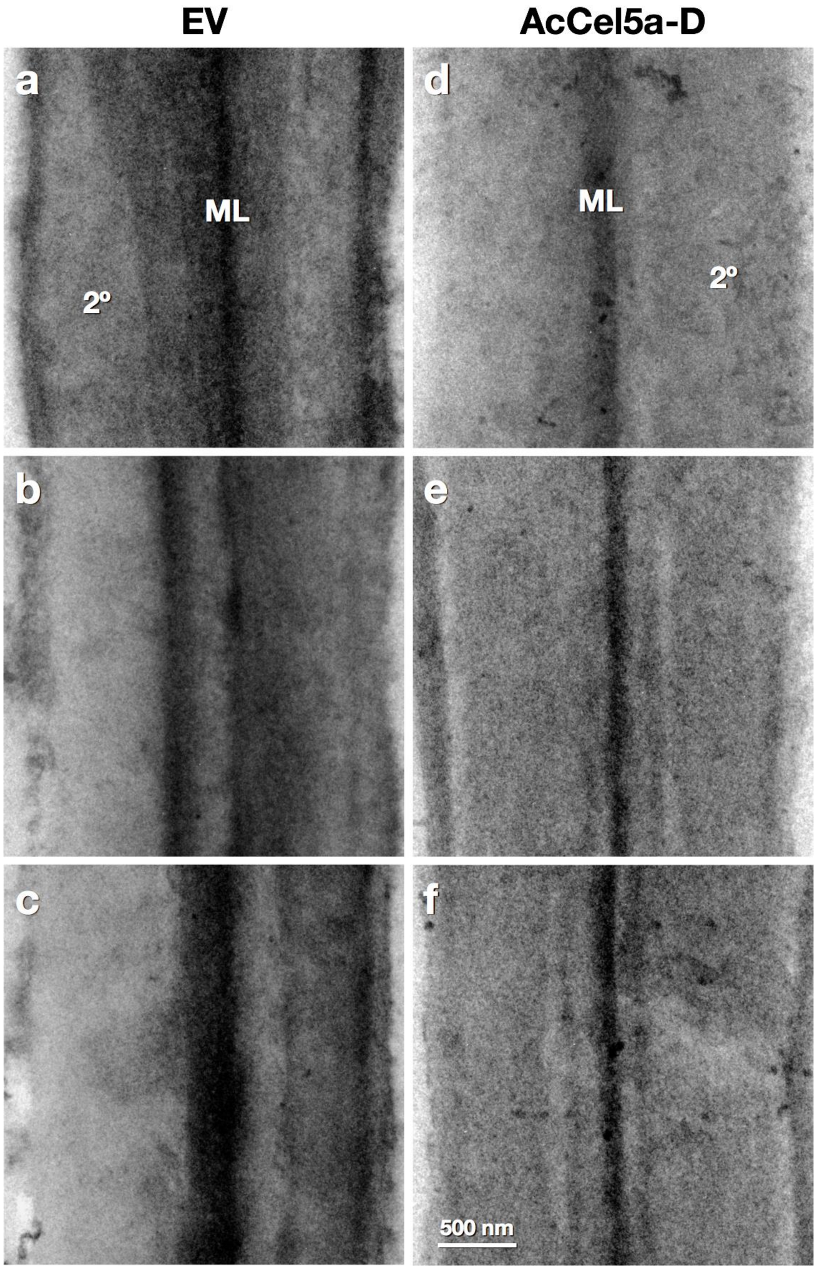


**Figure S4.** Gallery of TEM micrographs of the EV and AcCel5a-D plant cell walls of interfascicular fiber cells display examples of wild-type cell wall morphologies. ML-middle lamella, 2ºCW-secondary cell wall. The AcCel5A-D data is from line 37-4-a.
